# Learning Curve, Training, and Operator Experience in Vaginoscopic Hysteroscopy: A Systematic Review

**DOI:** 10.7759/cureus.118081

**Published:** 2026-10-17

**Authors:** Guy lambert M Monzango Sibo, Phaka Phoba

**Affiliations:** 1 Gynecology, University of Kinshasa, Kinshasa, COD; 2 Obstetrics and Gynaecology, Cliniques Universitaires de Kinshasa, Kinshasa, COD

**Keywords:** clinical competence, lc-cusum, learning curve, office hysteroscopy, outpatient hysteroscopy, simulation, training, vaginoscopy

## Abstract

Vaginoscopic hysteroscopy, also known as the no-touch technique, is widely used in outpatient gynecologic practice, but the experience required to achieve reproducible competence remains uncertain. This systematic review synthesized clinical and simulation-based evidence on learning curves, training, operator experience, technical performance, and competency assessment in vaginoscopic hysteroscopy. Eligible reports explicitly described vaginoscopic or no-touch access and evaluated at least one learning-related outcome. The included studies differed substantially in design, operator experience, training context, and definitions of technical performance. Clinical reports suggested improvement in procedural efficiency and success with experience, while simulation-based studies identified differences in technical performance between operators of varying experience. However, procedural speed, overall outpatient hysteroscopy proficiency, and vaginoscopy-specific competence were not assessed consistently, and the available evidence did not establish a validated, universal case-number threshold for independent vaginoscopic cervical access. No included clinical study established an individualized competency threshold for vaginoscopic access using sequential learning-curve methodology. These findings favor multidimensional, performance-based assessment rather than reliance on procedure counts alone and support prospective studies with standardized success criteria and individualized monitoring.

## Introduction and background

Office hysteroscopy is an established method for investigating and treating intrauterine pathology. The vaginoscopic approach, often called the no-touch technique, advances the hysteroscope through the vagina and cervical canal without routine insertion of a speculum or use of a cervical tenaculum. Compared with the traditional approach, vaginoscopy has been associated with lower pain, shorter procedure duration, and fewer vasovagal episodes without a clear increase in failure rates [1]. The 2024 Royal College of Obstetricians and Gynaecologists guideline recommends vaginoscopy as the standard approach for outpatient hysteroscopy unless a speculum is required for a specific clinical reason [2].

The technique nevertheless requires distinct psychomotor skills. The hysteroscopist must maintain orientation in a distended vaginal cavity, identify the external cervical os, align the instrument with the cervical canal, advance under direct vision, and enter the uterine cavity without unnecessary cervical instrumentation. Bettocchi and Selvaggi [3] helped establish this technique in clinical practice in the 1990s, reporting 1,200 office hysteroscopies, with the last 680 performed using the vaginoscopic approach without preselection.

Although the technique is now widely accepted, its educational requirements are poorly standardized. A 2016 systematic review of hysteroscopy training found substantial heterogeneity in simulators, curricula, technical outcomes, and assessment tools, with limited validity evidence for many competence measures [4]. A more recent systematic review including 39 studies reached a similar conclusion: simulation improves hysteroscopic performance, but optimized training pathways and robust pass-fail criteria remain insufficiently defined [5].

For vaginoscopy specifically, the clinically important question is not simply whether performance improves with experience, but when a trainee can be considered competent and how that competence should be measured. A fixed case number may be attractive for credentialing, but it may not account for individual learning rates, procedure complexity, need for supervisor assistance, or safety outcomes.

The primary objective of this systematic review was therefore to synthesize the available evidence on the learning curve and competency acquisition in vaginoscopic hysteroscopy. Secondary objectives were to examine the effect of operator experience on technical performance, identify the outcomes used to assess learning, evaluate the contribution of simulation and structured assessment, and determine whether the evidence supports a universal minimum number of procedures for competency.

## Review

Methods

This systematic review was developed and reported according to the Preferred Reporting Items for Systematic Reviews and Meta-Analyses (PRISMA) 2020 framework [6]. A review protocol defining the research question, eligibility criteria, outcomes, and planned search concepts was developed before final study selection, but it was not prospectively registered.

The review question was: among physicians learning or performing hysteroscopy by a vaginoscopic or no-touch approach, how does technical performance change with experience, and what evidence defines the number of procedures or performance threshold required to achieve competence?

Eligible reports included residents, fellows, gynecologists, or other physicians learning or performing vaginoscopic hysteroscopy. Clinical studies and simulation studies were considered. The vaginoscopic, no-touch, or speculum-free approach had to be explicit in the report, or the method had to clearly demonstrate atraumatic vaginal and cervical entry without routine speculum-assisted access. At least one learning-related outcome was required, including sequential performance, cumulative experience, operator experience, procedure time, technical success, failure, conversion to a speculum-assisted approach, supervisor assistance, technical assessment score, or a predefined competency threshold.

Randomized trials, prospective and retrospective cohorts, service audits, learning-curve studies, simulation-validation studies, and published conference abstracts with sufficient methodological information were eligible. Studies were excluded when they evaluated conventional hysteroscopy only, operative hysteroscopy without assessment of vaginoscopic access, pain or feasibility without any learning or operator-experience variable, or generic hysteroscopy simulation that did not reproduce vaginoscopic access. Reviews, guidelines, and commentaries were used for citation tracking and contextual discussion but were not included as primary studies.

Search concepts combined hysteroscopy terms with vaginoscopy/no-touch/speculum-free terms and learning-related terms such as learning curve, training, competence, proficiency, skill, experience, operator, trainee, resident, performance, simulation, cumulative sum, and learning-curve cumulative sum. Searches and citation tracking covered the literature from database inception through June 30, 2026. MEDLINE/PubMed was used as the principal biomedical database, and complementary searches were undertaken across Embase, Scopus, Web of Science, CENTRAL, CINAHL, conference proceedings, backward reference screening, and forward citation tracking. The final Boolean strategy was adapted to each database syntax.

Screening was deliberately restrictive. Reports of mini-hysteroscopy or outpatient hysteroscopy were not automatically classified as vaginoscopy; the method had to confirm the relevant mode of entry. Both authors participated in refinement of the search keywords, literature searching, and selection of candidate studies. Candidate records and potentially eligible full reports were reviewed collaboratively, and inclusion decisions were reached by discussion, with final eligibility and bibliographic verification performed by the corresponding author. The screening process was collaborative rather than conducted as two blinded, independent parallel reviews, and this is acknowledged as a methodological limitation.

For each eligible study, the following information was extracted when available: year, country, study design, number and experience of operators, number of patients or procedures, type of vaginoscopic technique, training intervention, definition of success, procedure time, pain outcomes, technical assessment instrument, need for assistance, learning threshold, and principal conclusion.

Risk of bias was assessed using validated design-specific Joanna Briggs Institute (JBI) critical appraisal tools. Pluchino et al. [7] was appraised using the revised JBI randomized controlled trial tool [8]. The JBI cohort checklist was applied to Di Spiezio Sardo et al. [9] and Miles et al. [10]. The JBI case-series checklist [11] was applied to Bettocchi and Selvaggi [3], Gkrozou et al. [12], and Keedwell et al. [13]. The JBI analytical cross-sectional checklist [14] was applied to Lau and Cheung [15]. Each domain was rated Yes, No, Unclear, or Not applicable. Unreported domains in conference abstracts were rated Unclear rather than assumed to be satisfied, and no numerical summary quality score was calculated.

A meta-analysis was not undertaken because the studies did not share a common definition of competency or sufficiently comparable learning outcomes. Clinical longitudinal learning evidence, cross-sectional experience-performance evidence, and simulation/competency evidence were therefore synthesized separately.

Core PubMed concept: (hysteroscop*) AND (vaginoscop* OR "no-touch" OR "no touch" OR "speculum-free" OR "speculum free") AND ("learning curve" OR learn* OR train* OR competen* OR proficien* OR skill* OR experienc* OR performan* OR operator* OR novice* OR trainee* OR resident* OR CUSUM OR "LC-CUSUM" OR simulat* OR assessment), with publication dates through June 30, 2026.

Results

Seven reports were considered directly relevant to learning, training, competency assessment, or operator experience in vaginoscopic hysteroscopy. Four were full journal articles and three were published conference abstracts. The evidence base extended from the initial clinical adoption of vaginoscopy in 1997 to dedicated vaginoscopy simulation and competency assessment reported in 2024.

The studies were heterogeneous in design and in the construct considered to represent learning. Two reports contained clear longitudinal clinical learning signals, two primarily evaluated relationships between operator experience and clinical performance, and three focused on broader training or simulation-based competency assessment. No included clinical study used cumulative sum (CUSUM) or learning-curve cumulative sum (LC-CUSUM) specifically to establish an individualized competency threshold for vaginoscopic cervical access.

Study selection and PRISMA flow

Across the reconstructed search branches documented in this review, 40 reference occurrences were identified. After removal of 7 duplicate occurrences, 33 unique records were screened. Fourteen records were excluded at the title/abstract stage. Nineteen reports were sought, and all were retrieved and assessed for eligibility, of which 12 were excluded after full-text or full-report evaluation (4 because no eligible vaginoscopic/no-touch access was demonstrated, 7 because no learning or operator-experience outcome was reported, and 1 because the accessible report did not provide sufficient learning-curve detail). Seven studies met the eligibility criteria and were included in the qualitative synthesis. No meta-analysis was performed because of marked methodological and clinical heterogeneity. Figure 1 summarizes this PRISMA 2020 study-selection process.

**Figure 1:**
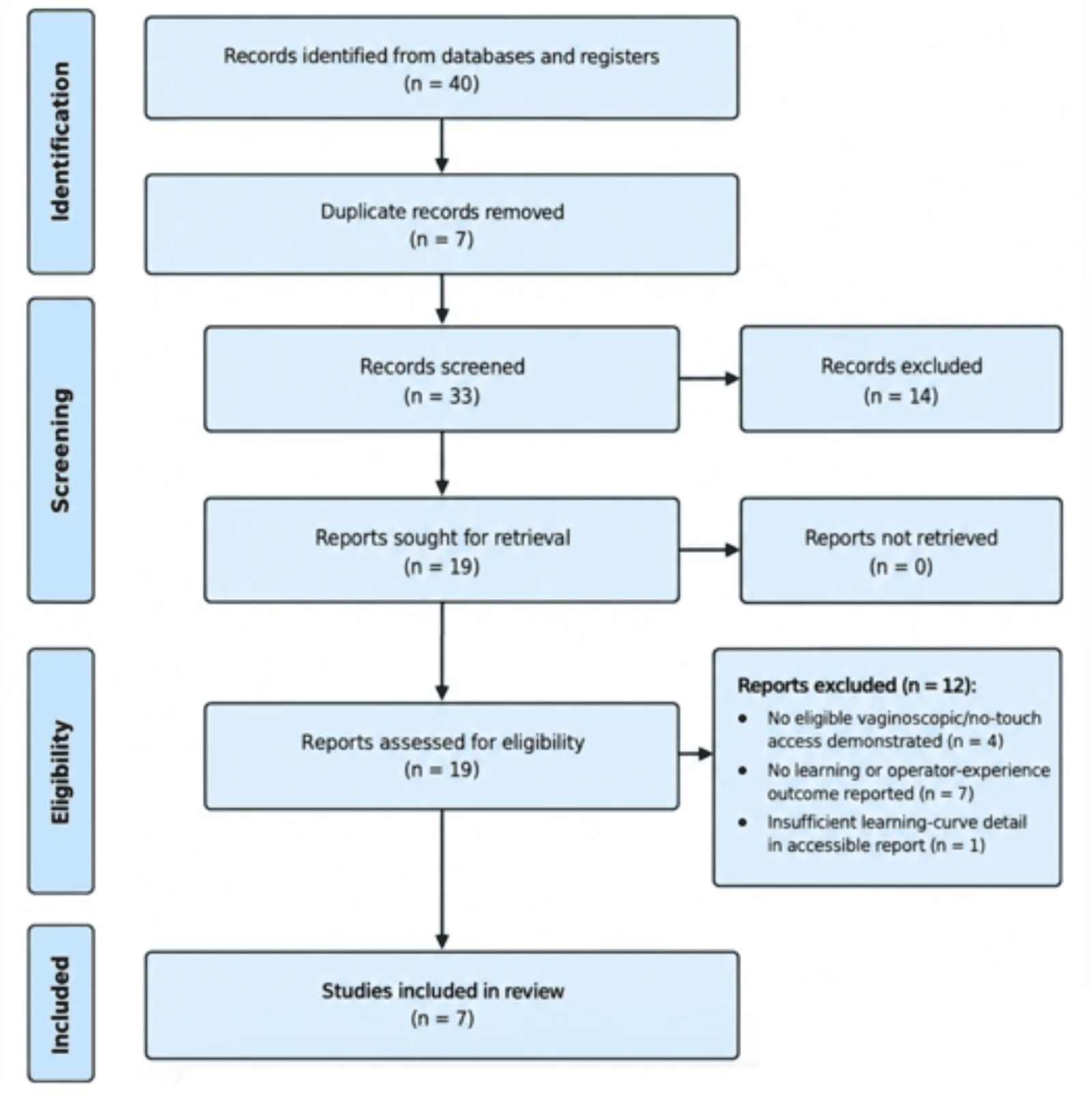
PRISMA 2020 flow diagram for study selection. Flow diagram based on the official PRISMA 2020 template [6]. The review identified 40 reference occurrences across the documented search branches; after the removal of 7 duplicates, 33 unique records were screened, 19 reports were assessed for eligibility, and 7 studies were included. Because source-specific identification counts could not be reconstructed from the available search records, the identification count is presented as an aggregate rather than estimated by source. PRISMA: Preferred Reporting Items for Systematic Reviews and Meta-Analyses

Table 1 summarizes the study design, sample size, vaginoscopic learning exposure, outcomes, principal findings, and interpretation of all seven included reports.

**Table 1:** Characteristics of the seven included reports. OSATS, Objective Structured Assessment of Technical Skills, originally described by Martin et al. [16]; VTSS-18, 18-item Vaginoscopy Task Simulator Scale; S-CVI/Ave, average scale content validity index; S-CVI/UA, scale content validity index with universal agreement

Study	Study design	Sample size	Vaginoscopic learning exposure	Outcome(s)	Main finding	Interpretation
Bettocchi & Selvaggi, 1997 [3]	Comparative clinical series	1,200 office hysteroscopies; last 680 vaginoscopic	Early adoption of no-touch entry	Feasibility and discomfort	The last 680 procedures were performed vaginoscopically without preselection; discomfort was reduced, including in women with moderate internal-os stenosis; the technique was described as easy to perform and without additional cost.	Foundational feasibility evidence; the primary report did not establish a formal learning or competency threshold.
Di Spiezio Sardo et al., 2008 [9]	Retrospective consecutive series	5,000 patients; 362 operators	Operators with different experience; traditional vs vaginoscopic insertion	Success, failure, complications	Nearly 95% success; traditional insertion and larger-caliber instrumentation were associated with greater failure, while high operator expertise was not a prerequisite for overall outpatient success.	Large real-world experience-performance evidence, but no individual longitudinal learning threshold.
Pluchino et al., 2010 [7]	Prospective randomized multicenter trial	184 women (92 conventional; 92 minihysteroscopy)	Hysteroscopists with varying experience	Pain, complications, satisfaction	Minihysteroscopy produced less pain, fewer complications, and greater satisfaction independent of hysteroscopist experience.	Experience was examined, but the trial did not define a learning curve or case-number threshold.
Gkrozou et al., 2015 [12]	Service audit / training report (conference abstract)	>1,200 outpatient procedures; vaginoscopy in 40%	Training of new outpatient hysteroscopists	Acquisition of overall outpatient skill set	Trainees required about 48 procedures to learn all techniques, including flexible and rigid vaginoscopy; authors emphasized individualized, competence-based attainment.	The 48 procedures represent the broader outpatient hysteroscopy skill set, not a vaginoscopy-specific threshold.
Keedwell et al., 2015 [13]	Longitudinal service evaluation (conference abstract)	>1,000 cases over 4 years	Clinicians initially inexperienced in outpatient hysteroscopy and vaginoscopy	Success and pain over time	Success increased from 87% to 98%; mean pain scores showed no learning-curve effect.	Strong service-level longitudinal learning signal; exact case-specific pass threshold not reported.
Miles et al., 2022 [10]	Prospective simulation cohort	30 physicians: 9 junior residents, 11 senior residents, 10 attendings	Experience-level comparison	Procedure time, modified Global Rating Scale, OSATS	Attendings were faster than junior residents and had higher technical ratings; senior residents also outperformed junior residents on Global Rating Scale scores.	Supports discriminant validity of the simulator; not a longitudinal clinical learning curve.
Lau & Cheung, 2024 [15]	Simulation / scale-validation study (conference abstract)	Participant number not reported in the abstract	Structured vaginoscopy training	VTSS-18 cognitive, psychomotor, and functional domains	VTSS-18 showed excellent content validity (S-CVI/Ave 0.98; S-CVI/UA 0.89) and high post-training domain scores.	Promising structured assessment; clinical transfer and pass-fail competency threshold remain unvalidated.

Longitudinal clinical learning

Bettocchi and Selvaggi [3] reported one of the foundational clinical experiences with the no-touch approach. Among 1,200 hysteroscopies performed between 1992 and 1996, the last 680 were completed using vaginoscopy without preselection. The primary report documented reduced discomfort, including in patients with moderate internal cervical-os stenosis, and described vaginoscopy as easy to perform, but it did not define a formal learning threshold. A later retrospective publication by Tsonis et al. [17] cited an approximate 10-case estimate, attributed to the early Bettocchi experience, for procedure duration to become comparable with traditional hysteroscopy. Because this estimate is a later interpretation rather than a validated competency endpoint in the primary report, it should be treated as a signal of early procedural familiarization rather than proof of global clinical competence.

The clearest longitudinal service-level evidence came from Keedwell et al. [13]. In a newly established ambulatory service, clinicians had no previous experience of outpatient hysteroscopy or vaginoscopy. Analysis of more than 1,000 cases over four years showed satisfactory success from the outset, increasing from 87% to 98%. In contrast, mean pain scores did not demonstrate a learning-curve effect. These findings suggest that technical success and patient experience can follow different learning trajectories and should not be treated as interchangeable markers of competence.

Operator experience and clinical performance

Di Spiezio Sardo et al. [9] analyzed 5,000 consecutive outpatient hysteroscopies performed by 362 operators with different levels of expertise. The mean number of procedures per operator was 13.8, and overall hysteroscopy was successfully completed in nearly 95% of cases. Traditional hysteroscope insertion, larger-caliber instrumentation, and several patient-related factors were associated with a greater risk of failure. Although this study did not follow individual operators sequentially, it provides large real-world evidence that high operator expertise was not a prerequisite for successful outpatient hysteroscopy and that vaginoscopic insertion was associated with fewer failures.

Pluchino et al. [7] studied 184 infertile women in a prospective multicenter randomized design and specifically included hysteroscopists with varying degrees of experience. Women were randomized to conventional hysteroscopy or minihysteroscopy, and discomfort, complications, and satisfaction were assessed. Minihysteroscopy was associated with lower pain, fewer complications, and greater satisfaction independent of hysteroscopist experience. The study therefore informs the role of experience as a clinical variable but does not identify a number of cases required for competence.

Training and simulation evidence

Gkrozou et al. [12] reported experience from more than 1,200 outpatient hysteroscopy procedures, with vaginoscopy used in 40% of cases. Trainees required approximately 48 procedures to learn the overall outpatient hysteroscopy skill set, including vaginoscopy with both flexible and rigid hysteroscopes. The authors emphasized that attainment of skill was individual and competence based. The 48-procedure estimate should therefore not be interpreted as a requirement for 48 vaginoscopies.

Miles et al. [10] developed a low-fidelity model specifically for office-based hysteroscopy using a vaginoscopic approach. Thirty physicians participated: nine junior residents, 11 senior residents, and 10 attending physicians. Attendings completed the simulation more rapidly than junior residents (197.2 ± 30.9 vs 289.8 ± 107.4 seconds, p=0.022). Attendings also had higher Global Rating Scale scores than junior residents (26.1 ± 2.4 vs 22.5 ± 3.7, p=0.01), and senior residents similarly outperformed junior residents. The model therefore discriminated between experience levels, but it did not measure a longitudinal clinical learning curve.

Lau and Cheung [15] introduced the VTSS-18, a dedicated 18-item scale spanning cognitive, psychomotor, and functional domains of vaginoscopy. The scale demonstrated excellent content validity, with an average scale content validity index of 0.98 and universal agreement of 0.89. Participants achieved high post-training scores across all domains. The work moves vaginoscopy assessment toward structured, observable performance, although the abstract did not report the participant number, and clinical transfer and a pass-fail case threshold remain unvalidated.

Number of procedures required for competency

The available literature does not establish a single universal number of procedures required for competency in vaginoscopic hysteroscopy. Two numerical estimates appear in the literature but represent different constructs. The approximately 10-case estimate is a later report of procedural-time stabilization attributed to early vaginoscopy experience [17], whereas the approximately 48-procedure estimate reflects acquisition of the broader outpatient hysteroscopy skill set, only part of which involved vaginoscopy [12].

Keedwell et al. [13] demonstrated progressive improvement in success without reporting the exact case number at which a stable plateau was reached. Miles et al. [10] and Lau and Cheung [15] demonstrated experience-related technical differences and structured competency measurement, respectively, but neither study followed trainees through repeated clinical cases to a predefined pass threshold.

Accordingly, averaging the available numerical estimates would be clinically misleading. The principal evidence gap is not the absence of any learning effect; rather, it is the absence of a validated definition of clinical competence and an individualized method for determining when that standard has been reached.

Table 2 summarizes the principal learning and competency signals across the evidence domains.

**Table 2:** Interpretation of the main learning and competency signals in the literature.

Evidence domain	Best available evidence	What can be concluded	What cannot be concluded
Initial technical familiarization	Bettocchi & Selvaggi [3]; later interpretation by Tsonis et al. [17]	Basic maneuvering may become more efficient relatively quickly.	Competence cannot be declared after 10 cases.
Clinical success over time	Keedwell et al. [13]	Service-level success improved with accumulated experience.	The exact case number to plateau is unknown.
Operator experience effect	Di Spiezio Sardo et al. [9]; Pluchino et al. [7]	Operator experience can be studied alongside technical and patient-related outcomes.	Cross-sectional or covariate analyses do not define individual learning curves.
Broader outpatient training	Gkrozou et al. [12]	Comprehensive outpatient hysteroscopy competence may require substantially more experience than basic entry skills.	The 48-procedure estimate is not a vaginoscopy-only threshold.
Simulation competency	Miles et al. [10]; Lau & Cheung [15]	Simulation can discriminate experience levels and structure assessment.	Clinical pass-fail validity and transfer remain insufficiently established.
Sequential competency monitoring	No vaginoscopy-specific included study	A major evidence gap remains.	No validated vaginoscopy-specific CUSUM/LC-CUSUM threshold can currently be recommended.

Risk of bias

Risk-of-bias assessments are presented by study design in Tables 3-6. The randomized trial by Pluchino et al. [7] had some concerns because allocation-concealment and blinding details were incompletely reported, and operator experience itself was not the randomized exposure. The observational full-text studies by Bettocchi and Selvaggi [3], Di Spiezio Sardo et al. [9], and Miles et al. [10] had mainly moderate concerns related to confounding or nonrandom allocation of technique and experience. The conference abstracts by Gkrozou et al. [12], Keedwell et al. [13], and Lau and Cheung [15] had greater uncertainty because several methodological domains could not be evaluated from the limited reports.

Table 3 presents the domain-specific JBI risk-of-bias assessment for the randomized controlled trial.

**Table 3:** JBI risk-of-bias assessment for the randomized controlled trial. JBI risk-of-bias assessment for the randomized controlled trial (Barker et al. [8]).

JBI domain	Pluchino et al. [7]	Rationale
1. True randomization used	Yes	Random allocation to conventional hysteroscopy or minihysteroscopy was reported.
2. Allocation concealment	Unclear	The accessible report did not provide sufficient detail.
3. Groups similar at baseline	Unclear	Baseline comparability was not sufficiently detailed in the accessible report.
4. Participants blinded	Unclear	Blinding was not clearly reported.
5. Treatment providers blinded	No	The hysteroscopist could not reasonably be blinded to instrument size/technique.
6. Outcome assessors blinded	Unclear	Blinding of outcome assessment was not clearly reported.
7. Groups treated identically apart from intervention	Yes	The comparison was otherwise conducted within the same trial framework.
8. Follow-up complete	Yes	Outcomes were assessed around the index procedure; no material attrition was reported.
9. Analyzed in randomized groups	Yes	The two randomized groups were reported as 92 women each.
10. Outcomes measured similarly between groups	Yes	Pain, complications, and satisfaction were measured in both groups.
11. Outcomes measured reliably	Yes	Pain was assessed using a visual analog scale and procedural outcomes were predefined.
12. Appropriate statistical analysis	Yes	Comparative analyses were reported for the randomized groups.
13. Trial design appropriate	Yes	The parallel randomized multicenter design was appropriate for the instrument comparison.
Overall judgment	Some concerns	Main concerns were incomplete reporting of concealment/blinding and the fact that operator experience was not itself randomized.

Table 4 presents the domain-specific JBI risk-of-bias assessment for the cohort studies.

**Table 4:** JBI risk-of-bias assessment for cohort studies. JBI risk-of-bias assessment for cohort studies (Moola et al. [14]).

JBI domain	Di Spiezio Sardo et al. [9]	Miles et al. [10]	Rationale
1. Groups similar / same population	No	No	Di Spiezio: technique depended on operator preference, experience, and patient characteristics; Miles: experience groups were inherently different.
2. Exposure measured similarly	Yes	Yes	Technique/experience categories were defined within each study.
3. Exposure measured validly/reliably	Yes	Yes	Insertion technique/instrument and training level were directly recorded.
4. Confounders identified	Yes	Unclear	Di Spiezio reported multiple patient and procedural factors; Miles did not fully characterize potential confounding.
5. Strategies to address confounding	Yes	No	Di Spiezio used multivariable association analyses; Miles mainly compared experience groups.
6. Participants free of outcome at exposure	Yes	Yes	Procedural/simulation outcomes occurred after the exposure classification.
7. Outcomes measured validly/reliably	Yes	Yes	Procedural success/failure and structured simulation ratings/timing were clearly measured.
8. Follow-up time sufficient	N/A	N/A	Outcomes were immediate procedural or single-session simulation outcomes.
9. Follow-up complete	Yes	Yes	The analyses used the procedural cohorts/simulation participants reported.
10. Strategies for incomplete follow-up	N/A	N/A	Not applicable to the immediate outcomes assessed.
11. Appropriate statistical analysis	Yes	Yes	Analyses were appropriate to the reported associations/comparisons.
Overall judgment	Some concerns	Some concerns	Main concerns relate to nonrandom exposure allocation/confounding; the Miles study was small and single-center.

Table 5 presents the domain-specific JBI risk-of-bias assessment for the case-series and service-evaluation reports.

**Table 5:** JBI risk-of-bias assessment for case-series/service-evaluation reports. For Gkrozou et al. [12] and Keedwell et al. [13], several domains were rated Unclear because only conference abstracts were available. For Bettocchi and Selvaggi [3], the accessible report provides limited information on participant characteristics and sampling methodology. JBI risk-of-bias assessment for case-series/service-evaluation reports (Munn et al. [11]).

JBI domain	Bettocchi & Selvaggi [3]	Gkrozou et al. [12]	Keedwell et al. [13]
1. Clear inclusion criteria	Unclear	Unclear	Unclear
2. Condition/procedure measured consistently	Yes	Unclear	Unclear
3. Valid identification methods	N/A	Unclear	Unclear
4. Consecutive inclusion	Unclear	Unclear	Unclear
5. Complete inclusion	Unclear	Unclear	Unclear
6. Demographics clearly reported	No	No	No
7. Clinical information clearly reported	Unclear	Unclear	Unclear
8. Outcomes/follow-up clearly reported	Yes	Yes	Yes
9. Site/setting clearly reported	Yes	Yes	Yes
10. Appropriate statistical analysis	Unclear	Unclear	Unclear
Overall judgment	High uncertainty	High uncertainty	High uncertainty

Table 6 presents the domain-specific JBI assessment for the analytical cross-sectional validation report.

**Table 6:** JBI risk-of-bias assessment for the analytical cross-sectional/validation report. JBI risk-of-bias assessment for the analytical cross-sectional/validation report (Moola et al. [14]).

	Lau & Cheung [15]	Rationale
1. Inclusion criteria clearly defined	Unclear	The conference abstract did not specify detailed participant inclusion criteria.
2. Subjects and setting described in detail	Unclear	The setting and varying experience were described, but participant number and detailed characteristics were not reported.
3. Exposure measured validly/reliably	Unclear	Years/levels of hysteroscopy experience were mentioned but measurement details were limited.
4. Objective standard criteria used	N/A	No diagnostic condition was being measured.
5. Confounders identified	No	Potential differences between experience levels were not formally addressed in the abstract.
6. Strategies to deal with confounding	No	No adjusted analysis was described.
7. Outcomes measured validly/reliably	Unclear	Strong content-validity indices were reported, but broader reliability/construct-validity details were not available in the abstract.
8. Appropriate statistical analysis	Yes	S-CVI/Ave and S-CVI/UA were appropriate for the reported content-validity analysis.
Overall judgment	High uncertainty	The abstract supports content validity but provides insufficient information for several other domains.

Discussion

The principal finding of this review is a mismatch between the clinical importance of vaginoscopy and the limited evidence defining how competence in the technique should be demonstrated. Vaginoscopy is recommended as the standard approach for many outpatient hysteroscopy procedures [2], yet no included study established a validated case-number threshold for independent clinical practice.

The frequently cited concept of a short learning curve should therefore be interpreted cautiously. The approximate 10-case estimate is reported in later literature as a procedural-time stabilization point attributed to early experience [17]; it does not incorporate supervisor intervention, failed entry, conversion to a speculum-assisted approach, safety events, quality of cavity visualization, or patient-centered outcomes. Speed is a useful technical marker, but speed alone is not synonymous with competence.

The Keedwell et al. [13] service data are especially informative because clinicians began without previous outpatient hysteroscopy or vaginoscopy experience and success improved substantially over time. The absence of a comparable learning effect on pain is equally important. Patient pain depends on operator technique but also on cervical anatomy, menopausal status, anxiety, instrument diameter, distention pressure, and the diagnostic or operative nature of the procedure. A competency framework should therefore include patient experience without using pain as the sole technical endpoint.

The 48-procedure estimate from Gkrozou et al. [12] further illustrates why case numbers should not be transferred uncritically between training contexts. These procedures represented a broad outpatient hysteroscopy curriculum involving multiple skills and instruments. The estimate is valuable as evidence that comprehensive outpatient practice requires broader experience than initial vaginoscopic entry, but it does not establish a vaginoscopy-specific credentialing number.

Evidence from outpatient hysteroscopy in general supports individualized monitoring. Issat et al. [18] applied LC-CUSUM to outpatient hysteroscopy and found that a third-year trainee reached a predefined performance standard after 56 procedures. That study was not specific to vaginoscopic access and was therefore not included in the primary synthesis, but it demonstrates the methodological advantage of LC-CUSUM: each trainee is evaluated according to the sequential pattern of successes and failures rather than being declared competent after an arbitrary fixed total. Although it did not evaluate a learning curve, the randomized study by Sharma et al. [19] provides relevant contextual evidence on the technical efficiency of the no-touch approach. Among 120 women, no-touch vaginoscopic hysteroscopy was significantly faster than the traditional technique (5.9 vs 7.8 minutes), with an overall hysteroscopy success rate of 99% and no significant difference in pain scores. These findings support the technical feasibility of vaginoscopy but do not define a learning threshold.

Individualized sequential performance monitoring using LC-CUSUM is particularly attractive for vaginoscopy because the technique contains discrete, measurable steps. Future studies could define success as identification of the external cervical os, atraumatic entry into the cervical canal, successful entry into the uterine cavity, absence of unplanned speculum or tenaculum use, absence of supervisor takeover, and completion within a clinically acceptable time. Secondary measures could include number of attempts, time to the external os, cervical access time, total procedure time, pain, vasovagal reactions, conversion, complications, and a structured technical score.

Simulation is likely to be most useful when integrated into such a competency-based pathway. The hysteroscopy education literature has repeatedly shown that simulation improves technical performance, especially among novices, while standardized pass-fail criteria and evidence of transfer to clinical practice remain limited [4, 5]. The vaginoscopy-specific model described by Miles et al. [10] and the VTSS-18 proposed by Lau and Cheung [15] provide practical foundations for structured preclinical training. Simulation can allow deliberate practice of navigation and cervical alignment before patient exposure, but independent clinical competence ultimately needs to be demonstrated in real procedures.

The implications for credentialing are direct. Current evidence does not justify requiring the same number of vaginoscopies from every trainee. A fixed-volume rule may undertrain slower learners and unnecessarily delay progression for faster learners. A more defensible pathway would combine standardized theory, video demonstration, simulation, supervised clinical procedures, and sequential performance monitoring until a predefined competency standard is met.

The findings also help explain why a technically simple procedure can still have a poorly characterized learning curve. Early success rates may already be high, reducing the statistical signal available to detect improvement. In addition, different outcomes mature at different speeds. Navigation may improve early, while consistency across difficult cervices, independent troubleshooting, and patient-centered performance may require more cases. A multidimensional definition of competence is therefore preferable to a single time threshold.

A prospective multicenter study specifically designed for vaginoscopic access is needed. Novice operators should be followed from their first supervised cases, using standardized instrumentation and predefined success and failure criteria. LC-CUSUM or related sequential methods could then estimate individual case numbers to competency and quantify interoperator variability. Such a study could also test whether prior endoscopic experience, simulation exposure, parity, menopausal status, cervical stenosis, or instrument diameter modifies the learning curve.

Strengths and limitations

A strength of this review is its specific focus on vaginoscopic access rather than hysteroscopy training in general. This distinction prevented generic operative hysteroscopy simulation studies from being interpreted as evidence about no-touch cervical entry. The review also separated longitudinal learning evidence from cross-sectional experience-performance comparisons and simulation-based competency evidence and added design-specific risk-of-bias appraisal.

The main limitation is the small and heterogeneous evidence base. Three of the seven directly relevant reports were published conference abstracts, limiting detailed appraisal of methods and operator-level data. Several studies compared operators with different experience rather than following the same trainee through consecutive cases. Older reports did not prospectively define competence, and terminology such as mini-hysteroscopy, office hysteroscopy, and vaginoscopy was not always used consistently.

A second limitation is that, although both authors participated in literature searching and study selection, screening was performed collaboratively rather than as duplicate independent review by two blinded reviewers. This may increase the possibility of selection or extraction error compared with fully independent duplicate screening. To reduce this risk, potentially eligible studies were discussed jointly, unclear reports were checked against full reports when available, and the corresponding author rechecked bibliographic details and key numerical findings against primary or published source records.

Risk-of-bias assessment was also constrained by reporting quality. In particular, the three conference abstracts did not provide enough information to judge several JBI domains, which were therefore classified as Unclear rather than assumed to be low risk.

Finally, quantitative pooling was not appropriate. The available numerical values represented different constructs, including procedural-time stabilization, broad outpatient skill acquisition, clinical success over time, and simulation performance. Combining them into a single average case number would create an estimate without a coherent clinical meaning.

## Conclusions

The available evidence indicates that technical performance in vaginoscopic hysteroscopy improves with experience and that basic maneuvering may be acquired relatively quickly. However, the literature does not support a universal minimum number of procedures for declaring clinical competence. The commonly cited early case estimates reflect different outcomes and training contexts rather than a validated pass threshold for independent vaginoscopic access.

Training should therefore move from simple procedure counts toward performance-based progression. A structured pathway combining theoretical preparation, simulation, supervised clinical practice, and individualized sequential monitoring is the most defensible approach based on current evidence. Prospective multicenter studies using predefined success criteria and LC-CUSUM or comparable sequential methods are needed to establish clinically meaningful competency thresholds.
